# Immunity‐modulated sex disparity on COVID‐19 prognosis

**DOI:** 10.1002/ctm2.164

**Published:** 2020-09-10

**Authors:** Dan Liu, Jiahao Liu, Shaoqing Zeng, Ya Wang, Yuan Yuan, Sen Xu, Siyuan Wang, Ruidi Yu, Xinxia Feng, Huayi Li, Xiaofei Jiao, Jianhua Chi, Chunrui Li, Fei Ye, Qinglei Gao

**Affiliations:** ^1^ National Medical Center for Major Public Health Events, Tongji Hospital, Tongji Medical College Huazhong University of Science and Technology Wuhan P. R. China; ^2^ Department of Gastroenterology Tongji Hospital, Tongji Medical College Huazhong University of Science and Technology Wuhan P. R. China; ^3^ Department of Hematology, Tongji Hospital, Tongji Medical College Huazhong University of Science and Technology Wuhan P. R. China; ^4^ Department of Neurosurgery, Tongji Hospital, Tongji Medical College Huazhong University of Science and Technology Wuhan P. R. China

Dear editor,

Coronavirus Disease 2019 (COVID‐19) is an emerging outbreak caused by the severe acute respiratory syndrome coronavirus 2 (SARS‐Cov‐2). It was first reported in Wuhan, China, and rapidly spread worldwide.^1^ Different studies have reported better survival advantages among female COVID‐19 patients than male patients.^2^ However, the underlying reasons for this disparity and sex‐specific influence of risk factors in disease progression remain unclear. More comprehensive studies are necessary, as it may help medical workers to lower the fatality rate for both sexes and better understanding of SARS‐CoV‐2.

Herein, we addressed this issue in a large‐scale, retrospective cohort study. A total of 2044 COVID‐19 patients (1044 females and 1000 males since January 27, 2020) from the Sino‐French New City campus and the Optical Valley Campus of Tongji hospital, were included in the final analysis (Supporting Information Methods and Figure S1). The clinical characteristics are demonstrated in Tables S1‐S3. Generally, female patients had better survival advantages. During COVID‐19 onset, female patients experienced less dyspnea and consciousness disorders. In addition, during hospitalization, better physical conditions such as fewer organ injuries and less immune dysfunction were documented in female patients (Figure S2). Consequently, corticosteroids, intravenous immunoglobin, broad‐spectrum antibiotics, and non‐invasive/invasive mechanical ventilations were required more for male patients compared with female ones.

For prognosis analysis, the maximum Sequential Organ Failure Assessment (SOFA) scores during patients’ hospitalization (SOFAmax) ≥5 were chosen as the primary endpoint, considering that SOFAmax ≥5 can reflect the severest multi‐organ dysfunctions during entire hospitalization and predict the in‐hospital death for patients with COVID‐19 directly (Figure S3).

The proportion of a higher SOFAmax increased with age in both sexes (Figure S4). SOFAmax was positively correlated with advanced age at a much higher extent in male patients than females (Figure S4). The effect modification between sex and age was statistically significant (*P* = .001, linear regression models).

On the contrary, multiple key factors reflecting cytokine storms (cytokine storms were characterized by elevated circulating levels of early response proinflammatory cytokines, such as tumor necrosis factor α [TNF‐α], interleukin [IL]‐6, IL‐2 receptor [IL‐2R], and IL‐8^3^, ^4^) and potential bacterial infections (elevated c‐reactive protein [CRP] and procalcitonin [PCT]) showed higher odds ratios for females than males. In female patients, the probability of developing SOFAmax ≥5 sharply increased to a much higher extent with the increase of these factors, when compared to males (Figure 1A‐D). The significant differences of ORs for IL‐6, IL‐2R, IL‐8, TNF‐α, CRP, and PCT between different sexes were confirmed using logistic regression models containing the interaction terms (sex and other variables; **Table** **1**). As sex was an effective modifier of the above‐mentioned important factors in disease progression, it was necessary to explore risk factors on a sex basis using multivariable analysis (**Table** **1**).

**FIGURE 1:**
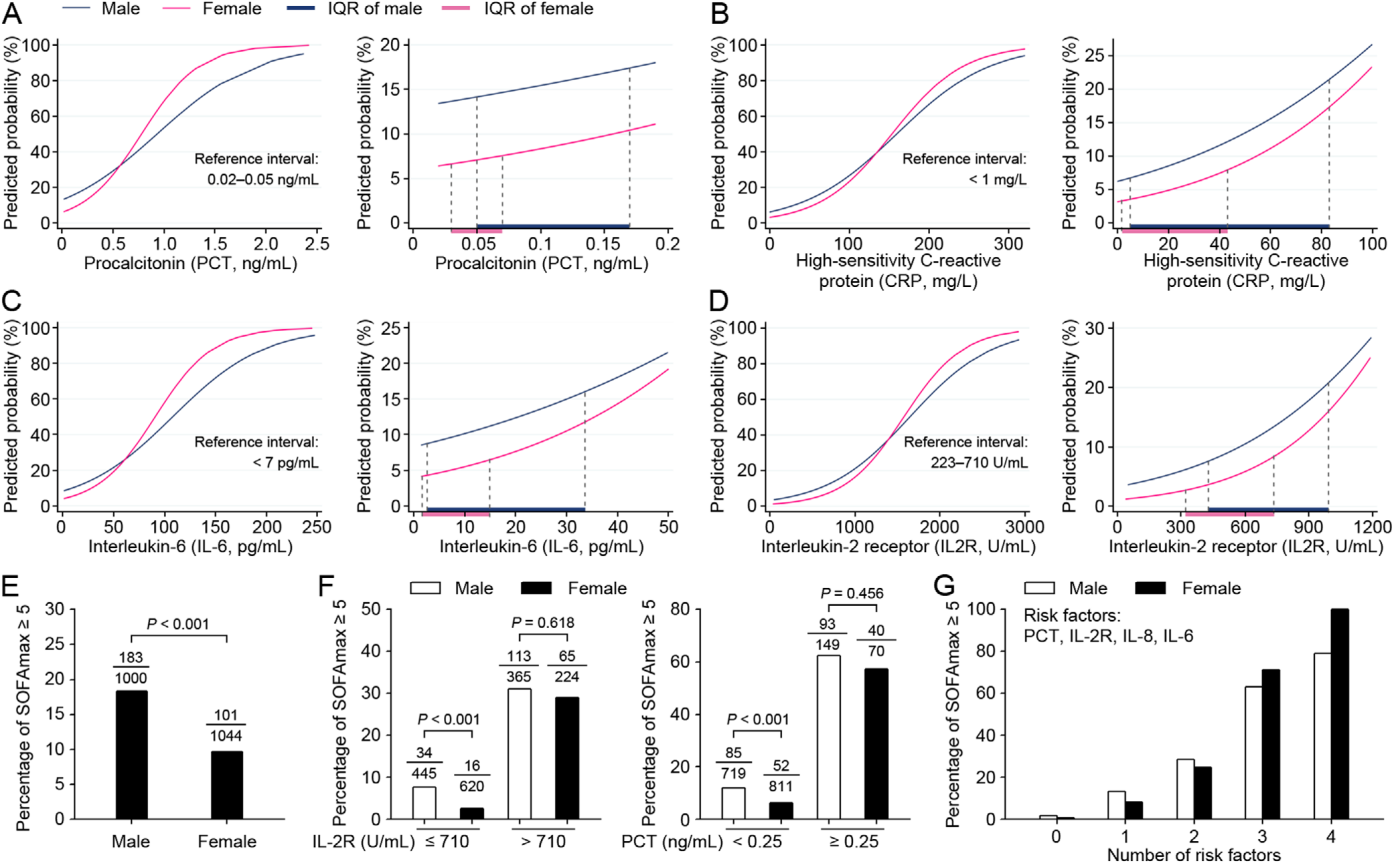
Different effects of multiple factors on multi‐organ injuries between male and female patients with COVID‐19. A–D, The probabilities of developing SOFAmax ≥5 were predicted using logistic regression models containing the interaction terms of sex and PCT (A), sex and CRP (B), sex and IL‐6 (C), sex, and IL‐2R (D), respectively. The IQR of each factor for male and female patients was shown in the right of the corresponding panel. E, The proportions of SOFAmax ≥5 for male and female patients with COID‐19. F. The proportions of SOFAmax ≥5 in male and female patients with different levels of IL‐2R and PCT. G. The proportions of SOFAmax ≥5 in male and female patients with a series number of risk factors. The risk factors include PCT ≥ 0.25 ng/mL, IL‐2R > 710 U/mL, IL‐8 ≥ 62pg/mL, IL‐6 ≥ 14pg/mL. SOFAmax = maximum sequential organ failure assessment score during hospitalization. PCT = procalcitonin. IL‐2R = interleukin‐2 receptor. IL‐6 = interleukin‐6. IL‐8 = interleukin‐8

**TABLE 1:** Risk factors associated with SOFAmax ≥5 in male and female patients

	Univariable Analysis					
**SOFAmax ≥5**	Male		Female			
**Risk Factors**	OR (95% CI)	*P*‐value	OR (95% CI)	*P*‐value	Fold difference of ORs between male and female^b^	Effect modification*P*‐value^a^
Age, > 50	11.912 (5.207, 27.251)	<.001	5.781 (2.323, 14.387)	<.001	2.061	.048
Hypertension	3.008 (2.156, 4.198)	<.001	1.407 (0.932, 2.126)	.105	2.138	.002
CHD	2.869 (1.849, 4.451)	<.001	1.549 (0.829, 2.894)	.170	1.852	.044
COPD	2.866 (0.926, 8.864)	.068	38.804 (4.294, 350.646)	.001	0.074	.017
Anemia	1.378 (0.921, 2.062)	.119	2.130 (1.372, 3.306)	.001	0.647	.014
Blood urea nitrogen, ≥10 mmol/L	21.928 (13.267, 36.241)	<.001	53.939 (25.657, 113.397)	<.001	0.407	.039
Creatinine, ≥110 μmol/L	5.509 (3.670, 8.269)	<.001	16.017 (8.330, 30.800)	<.001	0.344	.004
Procalcitonin, Per 1 ng/mL increase	31.786 (14.450, 69.922)	<.001	33.246 (12.921, 85.542)	<.001	0.956	.010
C‐reactive protein, Per 1 mg/L increase	1.017 (1.014, 1.020)	<.001	1.022 (1.018, 1.027)	<.001	0.995	.044
IL‐6, Per 1 pg/mL increase	1.023 (1.018, 1.027)	<.001	1.036 (1.027, 1.044)	<.001	0.987	.007
IL‐2R > 710 U/mL	5.421 (3.582, 8.202)	<.001	15.432 (8.691, 27.404)	<.001	0.351	.005
IL‐8, ≥62 pg/mL	4.895 (2.875, 8.336)	<.001	9.908 (5.264, 18.647)	<.001	0.494	.025
TNFα, ≥8.1 pg/mL	2.994 (1.989, 4.507)	<.001	6.347 (3.746, 10.754)	<.001	0.472	.018

Abbreviation: OR, Odds ratio; CI, confidence interval; CHD, coronary heart disease; COPD, chronic obstructive pulmonary disease; IL2R, interleukin‐2 receptor; IL‐6, interleukin‐6; SOFAmax, maximum sequential organ failure assessment score during hospitalization.

^a^ Test of effect modification between sex and other factors using logistic regression models containing the interaction terms after adjusted by age.

^b^ Fold difference of OR between male and female patients is calculated using the following formula: OR of male/OR of female.

The sex‐specific influence on inflammatory biomarkers and cytokine levels were further analyzed. The majority of females had significantly lower levels of IL‐6, IL‐2R, IL‐8, TNF‐α, CRP, and PCT than males (Table S1 and Figures S1 and S5), resulting in a generally lower risk of developing SOFAmax ≥5 (Figure 1E). However, increased inflammation mediators dramatically increased the risk of SOFAmax ≥5 in females to the levels of males (Figure 1F). Additionally, for the four representative risk factors (including PCT ≥0.25 ng/mL, IL‐2R > 710U/mL, IL‐8 ≥62pg/mL, and IL‐6 ≥14pg/mL), co‐existence of three or four indexes corresponded to 71% or 100% of females who developed SOFAmax ≥5, respectively (Figure 1G). These results indicated a generally better prognosis for female patients than male ones, but a higher risk of poor prognosis for the females in hyperinflammatory states.

Overall, over‐reacted immune responses were found to impose different influences between sexes and bring more dangers to female patients. It seemed like a contradiction that the out‐of‐proportion inflammatory response to the virus was more injurious for female patients while they have better outcomes than males. In fact, the different prognoses between female and male COVID‐19 patients were produced by the co‐effect of different odds ratios and exposure levels of risk factors between sexes. The concentrations of inflammatory mediators and cytokines were lower in female patients than in males during their whole hospitalization cycle, resulting in their survival advantages. However, once immune dysfunctions happened, all survival advantages of females were diminished and more ferocious organ injuries were induced, which might result in the lethality of COVID‐19 in female patients. The dysfunctions happened in both the innate and adaptive immune systems, which were characterized as elevated TNF‐α, IL‐6, IL‐2 receptor, and IL‐8. Among them, TNF‐α can lead to macrophage activation, which contributes to hemophagocytic lymphohistiocytosis and anemia.^3^ IL‐6 could enhance the antigen stimulation with overproduction of pro‐inflammatory cytokines by inducing failure of cytotoxic T lymphocytes and natural killer cells.^4^ IL‐2 receptor is expressed by T cells and released into the bloodstream during immune activation.^5^ IL‐8 is a classic neutrophil chemoattractant and product of activated neutrophils.^6^

A higher “set‐point” of the innate immune system in females may explain the above‐mentioned differences. Compared to males, females have more efficient antigen­presenting cells and higher expressions of Toll­like receptor (TLR)­pathway and pro­inflammatory genes, making their inflammatory system more responsive. Responsive immunity may impose a double‐edged influence. At the early stage of viral infections, a controllable inflammatory response will be easily activated in females than in males, ensuring a rapid clearance of the virus and resolving the infection. However, when the virus is not cleared timely, a greater quantity of viruses could easily cause a significantly overreacted immune response and trigger a cytokine storm. Afterward, cytokines storms could lead to a variety of infectious and immune‐mediated conditions through organ systems from the lung to blood vessels with devastating consequences.^3^ Meanwhile, the X chromosome‐located immunoregulating genes and different immunomodulatory functions of sex hormones also contribute to the differential regulation of immune responses between the sexes.^7^ The above mechanisms need to be testified in future clinical and experimental studies.

Now, owing to the worldwide shortage in medical supplies and a higher risk of poor prognosis identified for male patients, medical workers might pay more attention to males who have more immune disturbance.^8^ However, our data suggest that overactivated immunity was more lethal in female COVID‐19 patients, and it is equally important that indications of immune dysfunctions should be closely monitored in female patients, to ensure the clinicians have the conversance on patient conditions and the promptitude on applying treatments accordingly. Otherwise, all survival advantages of female patients would be easily diminished and their conditions would deteriorate more rapidly than male patients.

According to previous studies, controlling inflammatory responses through immunomodulators (ie, corticosteroid, intravenous immunoglobulin, Tocilizumab, Baricitinib) are effective measures to improve the prognosis of COVID‐19. However, the efficiency of immune‐modulatory treatment on COVID‐19 has not been investigated via a sex‐based approach.^9^ Considering the sex‐specific influence of immunity, more studies are warranted in the future. Meanwhile, remdesivir, an RNA‐Dependent RNA Polymerase, was reported to achieve a reduction in time to recovery and a trend toward lower mortality among patients with COVID‐19.^10^, ^11^ Published data showed that the recovery rate ratio for female patients using remdesivir is slightly higher than male ones; however, whether this difference is statistically significant was not mentioned.^12^ So, we think that will be appropriate to make further analyses based on sexes.

The major limitation of this study is its retrospective nature. Medical information was collected from existing health care records. Thus, not all potential risk factors were available for analysis. For example, smoking predisposition, which is more prevalent in male patients, may also play a vital role in this interaction, as it worsens the prognosis of COVID‐19 by modulating the expression of ACE2 (the receptor for severe acute respiratory syndrome coronavirus 2 [SARS‐CoV‐2]).^13^ So, more investigations are warranted in the future.

In conclusion, frangible survival benefits in female COVID‐19 patients than male ones could be explained by their different clinical courses and the sex‐specific influences of risk factors. Out‐of‐proportion immune responses were highlighted as risk factors for both sexes with different effects. These were more prevalent in male patients but had greater influences on females. Thus, it is equally necessary that indications of immune disturbance and cytokine storms should be closely monitored in both sexes, to ensure timely treatments. In the future, more studies are warranted to explore mechanisms for the sex‐specific influence of risk factors.

## ETHICAL STATEMENT

This study was reviewed and approved by the Medical Ethical Committee of Tongji Hospital of Huazhong University of Science and Technology with waived informed consent (TJ‐IRB20200406) and registered at Chinese Clinical Trial Registry (No. ChiCTR2000032161).

## AUTHOR CONTRIBUTIONS

D Liu, J Liu, S Zeng, Y Wang, Y Yuan, and S Xu contributed equally to this work. D Liu, J Liu, and S Zeng collected the clinical data. Y Wang, Y Yuan, and S Xu analyzed the clinical records. D Liu analyzed and interpreted the data. J Liu and H Li drafted the manuscript. S Wang, R Yu, X Feng, X Jiao, and J Chi double‐checked and loaded the data into the database. C Li Advised on the conception and design of the study. Q Gao and F Ye are corresponding authors of this article. Q Gao conceptualized and designed the study, supervised the project, and revised the manuscript. F Ye conceptualized and designed the study, supervised the project, analyzed the data, and revised the manuscript. All authors vouch for the respective data and analysis, revised, approved the final version, and agreed to publish the manuscript.

## FUNDING INFORMATION

Fundamental Research Funds for the Central Universities: 2019kfyXMBZ024; National Science and Technology Major Sub‐Project: 2018ZX10301402‐002; Technical Innovation Special Project of Hubei Province: 2018ACA138; National Natural Science Foundation of China: 81572570, 81772787, 81873452, 81974405.

## CONFLICT OF INTEREST

All authors declare no competing interests.

## Supporting information

Supplement Figure 1: The flowchart of patient enrollment.
Supplement Figure 2: Dynamic changes in inflammation markers and cytokines from illness onset in male and female patients with COVID‐19.
Supplement Figure 3. Performance of SOFAmax in predicting the mortality of coronavirus disease 2019 patients.
Supplement Figure 4: Different effects of age on developing a higher SOFAmax score for male and female patients.
Supplement Figure 5: Difference of inflammation biomarkers and cytokines between female and male patients with COVID‐19.
Supplement Table 1. Clinical characteristics of male and female patients at admission.
Supplement Table 2. Treatments and outcomes of female and male patients.
Supplement Table 3. Prognosis analysis for female and male patients with COVID‐19.
Supplement Table 4. Risk factors associated with SOFAmax ≥5 in male and female patients revealed via univariable analysis.
Supplement Table 5. Risk factors associated with SOFAmax ≥5 in male and female patients revealed via multivariable analysis.

## Data Availability

The data that support the findings of this study are available from the corresponding author upon reasonable request.
