# Supplementary material for: Immunity‐modulated sex disparity on COVID‐19 prognosis
Source: Clin Transl Med. 2020 Sep 10;10(5):e164. doi: 10.1002/ctm2.164 (PMC7507394; doi:10.1002/ctm2.164)
Supplement: Supplementary file 1 — Supplement Figure 1: The flowchart of patient enrollment. Supplement Figure 2: Dynamic changes in inflammation markers and cytokines from illness onset in male and female patients with COVID‐19. Supplement Figure 3. Performance of SOFAmax in predicting the mortality of coronavirus disease 2019 patients. Supplement Figure 4: Different effects of age on developing a higher SOFAmax score for male and female patients. Supplement Figure 5: Difference of inflammation biomarkers and cytokines between female and male patients with COVID‐19. Supplement Table 1. Clinical characteristics of male and female patients at admission. Supplement Table 2. Treatments and outcomes of female and male patients. Supplement Table 3. Prognosis analysis for female and male patients with COVID‐19. Supplement Table 4. Risk factors associated with SOFAmax ≥5 in male and female patients revealed via univariable analysis. Supplement Table 5. Risk factors associated with SOFAmax ≥5 in male and female patients revealed via multivariable analysis. [file CTM2-10-e164-s001.docx]

**Supplementary**

**Supplement Methods**

**Supplement Method 1. Study design and participants**

**Supplement Method 2. Data collection**

**Supplement Method 3. Statistical analysis**

**Supplement Method 4. Laboratory confirmation of SARS-CoV-2**

**Supplement Method 5. Clinically diagnosed COVID-19**

**Supplement Method 6. Criteria for discharge of COVID-19 patients**

**Supplement Method 7. Disease severity of COVID-19**

**Supplement Method 8. Definitions of symptoms and clinical outcomes**

**Supplement Figures**

**Supplement figure 1: The flowchart of patient enrollment.**

**Supplement figure 2: Dynamic changes in inflammation markers and cytokines from illness onset in male and female patients with COVID-19.**

**Supplement figure 3. Performance of SOFAmax in predicting the mortality of coronavirus disease 2019 patients.**

**Supplement Figure 4: Different effects of age on developing a higher SOFAmax score for male and female patients.**

**Supplement figure 5: Difference of inflammation biomarkers and cytokines between female and male patients with COVID-19.**

**Supplement Tables**

**Supplement table 1. Clinical characteristics of male and female patients at admission.**

**Supplement table 2. Treatments and outcomes of female and male patients.**

**Supplement table 3. Prognosis analysis for female and male patients with COVID-19.**

**Supplement table 4. Risk factors associated with SOFAmax ≥5 in male and female patients revealed via univariable analysis.**

**Supplement table 5. Risk factors associated with SOFAmax ≥5 in male and female patients revealed via multivariable analysis.**

**Supplement Methods**

**Supplement Method 1. Study design and participants**

This retrospective cohort study enrolled all inpatients diagnosed with COVID-19 in the Sino-French New City campus since January 27, 2020, and the Optical Valley Campus of Tongji hospital since February 10, 2020, when the 40 national medical teams took over these two hospitals. 2387 patients with a definite clinical outcome (death or discharge) before March 21, 2020 were consecutively enrolled for further screening. Among them, 72 patients were excluded because of the absence of COVID-19 diagnosis, together with 205 patients who were transferred from mobile cabin hospitals for isolation, 31 patients who died within 24 hours of admission lacking blood test and medical history, and 35 patients who were under 18 years old, pregnant, re-hospitalized or discharged for dialysis 12. Finally, 2044 patients with COVID-19 were included. The flowchart of patient enrollment is demonstrated in **supplement figure 1**.

**Supplement Method 2. Data collection**

Clinical data including deidentified patient information, clinical symptoms and signs, nursing records, laboratory findings, interpretations on chest X-ray or compute tomography (CT), treatments, and patient outcomes, were extracted from electronic medical records using a standardized data collection form and stored in the database of Tongji Hospital for further analysis 4. The Sequential Organ Failure Assessment (SOFA) scores were measured for all patients and maximum SOFA scores during patients’ hospitalization (SOFAmax) ≥5 was used as the outcome event for prognosis analysis. All clinical data were reviewed by two trained physicians and a third researcher were involved if there is any difference in data interpretations. Definitions for patient conditions is in accordance with the previous studies and listed in the **supplement methods 7 and 8**.

**Supplement Method 3. Statistical analysis**

The sample size of this study equals to the number of hospitalized COVID-19 patients who were dead in-hospital or discharged during the study period. Descriptive statistics were obtained for all study variables. Continuous variables are presented as median and interquartile range (IQR). Categorical variables are expressed as number and percentage of patients. Mann-Whitney U test, χ² test, or Fisher’s exact test were used to compare the difference of clinical characteristics, treatments and occurrence rate of outcomes between male and female patients under different context. Univariable and multivariable logistic regression models were used to estimate the influence of sex on different complications and prognoses. Univariable and multivariable logistic regression models were also applied to explore the risk factors associated with the SOFAmax ≥5 for male and female patients separately. The odds ratio (OR) along with the 95% confidence interval (95%CI) was reported. The effect modification between sex and other factors were also tested using logistic regression models.

A two-sided α of less than 0.05 was considered statistically significant. Statistical analyses were done using the STATA software (version 15.1).

**Supplement Method 4. Laboratory confirmation of SARS-CoV-2**

Tongji Hospital was officially approved by the National Health Commission for confirmation of diagnosis. The reverse-transcription polymerase chain reaction (RT-PCR) assay was carried out according to the protocol recommended by the World Health Organization. Commercialized RNA extraction kits (Biogerm, Shanghai, China) were used to extract total RNA. Two targets were used in real time RT-PCR. Target 1: open reading frame 1 ab (ORF1ab), forward primer: 5′-CCCTGTGGGTTTTACACTTAA-3′, reverse primer: 5′-ACGATTGTGCATCAGCTGA-3′, and probe: 5'-VIC-CCGTCTGCGGTATGTGGAAAGGTTATGG-BHQ1-3'. Target 2: For the N region, forward primer: 5′-GGGGAACTTCTCCTGCTAGAAT-3′, reverse primer: 5′-CAGACATTTTGCTCTCAAGCTG-3′, and the probe: 5’-FAM-TTGCTGCTGCTTGACAGATT-TAMRA-3’. The real time RT-PCR assay was carried out with a SARS-CoV-2 nucleic acid detection kit according to the manufacturer’s protocol (Shanghai Bio-germ Medical Technology company). The exact protocol was previous reported ^1^.

**Supplement Method 5. Clinically diagnosed COVID-19**

The clinically diagnosed cases were only allowed for the cases in the Hubei Province for the period of February 9 to 19 based on the Diagnosis and Treatment guidance of COVID-19 (5^th^ edition) released by the National Health Commission of China on February 8, 2020 and abolished on February 19, 2020. A presumptive case was defined as meeting the following criteria: (1) recent travel history to Wuhan City or Hubei Province; or close contact with a confirmed or probable case; or cluster transmission; (2) fever and/or respiratory symptoms; (3) laboratory evidence of normal or decreased number of leukocytes and/or lymphopenia. Those presumptive cases with further radiographic evidence showing pneumonia but without a positive RT-PCR test result were defined as clinically diagnosed cases ^2^.

**Supplement Method 6. Criteria for discharge of COVID-19 patients**

The criteria for discharge were absence of fever for at least 3 days, substantial improvement in both lungs in chest computed tomography (CT), clinical remission of respiratory symptoms, and two throat-swab samples negative for SARS-CoV-2 RNA obtained at least 24 hours apart according to the Diagnosis and Treatment Scheme for COVID-19 released by the National Health Commission of China ^3^.

**Supplement Method 7. Disease severity of COVID-19**

The severity of COVID-19 was obtained in accordance with the 7th edition of the Diagnosis and Treatment Protocol of COVID-19 by the National Health Commission patients in moderate group have symptoms including fever and respiratory tract symptoms, and an imaging finding of pneumonia, but fails to meet the criteria for severe group and critical group. COVID-19 patients that meet any of the following criteria belong to severe group: 1) dyspnea or respiratory rate ≥ 30 breaths/min; 2) SPO2 ≤ 93·00% at a rest state; 3) arterial partial pressure of oxygen (PaO2)/oxygen concentration (FiO2) ≤ 300 mmHg; 4) patients with > 50·00% lesion progression in lung imaging within 48 hours. Critical group is defined as patients that meet any of the following criteria: 1) presence of respiratory failure and mechanical ventilation is required; 2) occurrence of shock; 3) complicated with other organ failure that requires admission to ICU.

**Supplement Method 8. Definitions of symptoms and clinical outcomes**

- Fever was defined as axillary temperature of at least 37·3°C.
- Sepsis and septic shock were defined according to the 2016 Third International Consensus Definition for Sepsis and Septic Shock ^4^.
- Acute kidney injury was diagnosed according to the KDIGO guidelines ^5^.
- Acute respiratory distress syndrome (ARDS) was diagnosed according to the Berlin Definition ^6^.
- Acute cardiac injury was diagnosed if serum level of cardiac troponin I was above the 99th percentile upper reference limit.
- Coagulopathy was defined as a 3-second extension of PT or a 10-second extension of APTT.
- Hypoproteinemia was defined as serum level albumin ≤ 30 g/L.
- SOFA score was referred to previous reported ^4^.
- Acute heart failure was diagnosed with typical symptoms (e.g. breathlessness) and accompanied signs (e.g. elevated jugular venous pressure) caused by a cardiac abnormality ^7^.
- Acute liver injury was defined as serum level of total bilirubin ≥ 3 mg/dl and an acute increase in ALT of at least five times the upper limit of the normal range and/or an increase in alkaline phosphatase of at least twice the upper limit of the normal ^1^.

**Supplement Figures**

**Supplement figure 1: The flowchart of patient enrollment.**


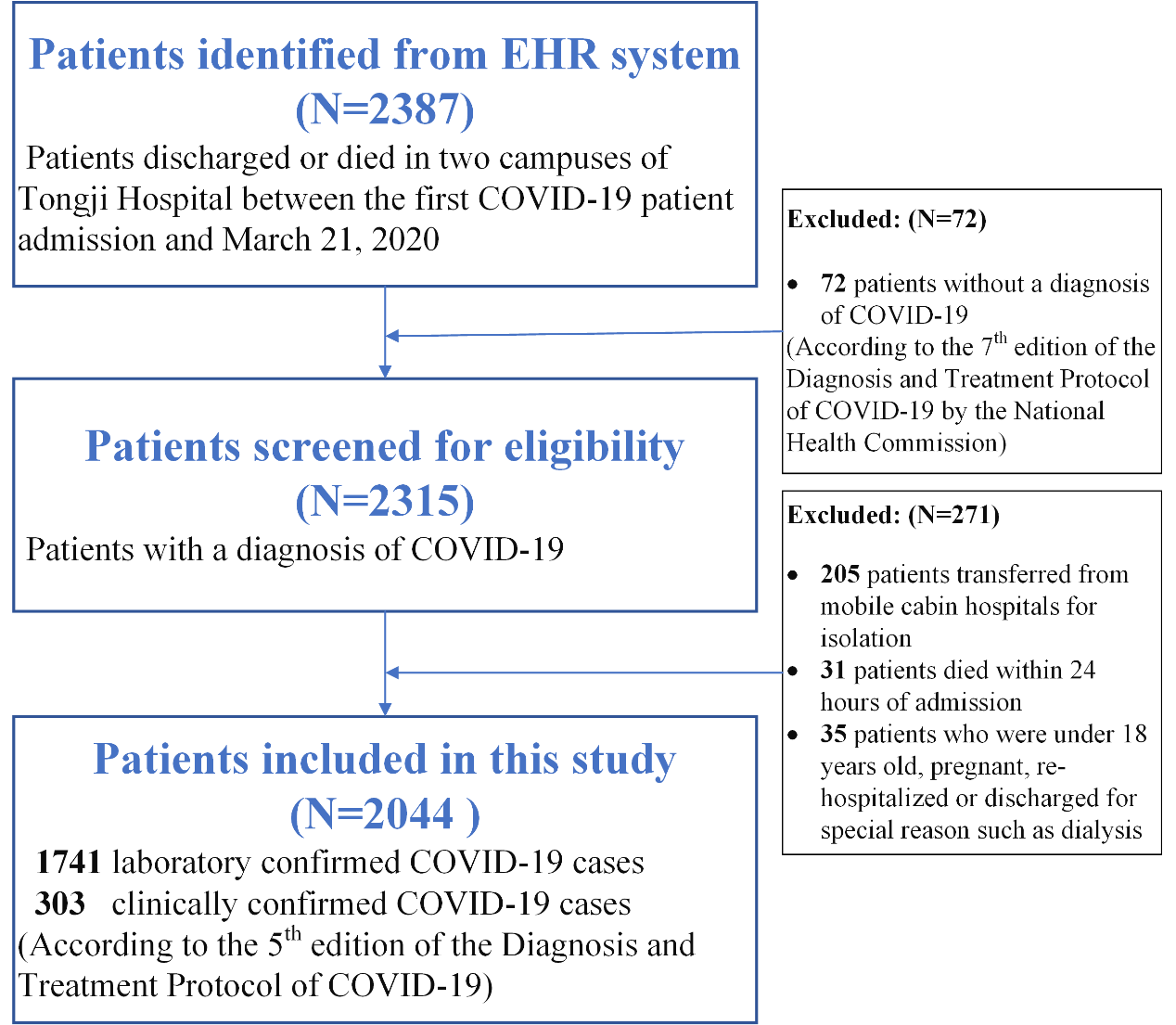


EHR=Electronic healthcare records. COVID-19=Coronavirus Disease 2019.

**Supplement figure 2: Dynamic changes in inflammation markers and cytokines from illness onset in male and female patients with COVID-19.**


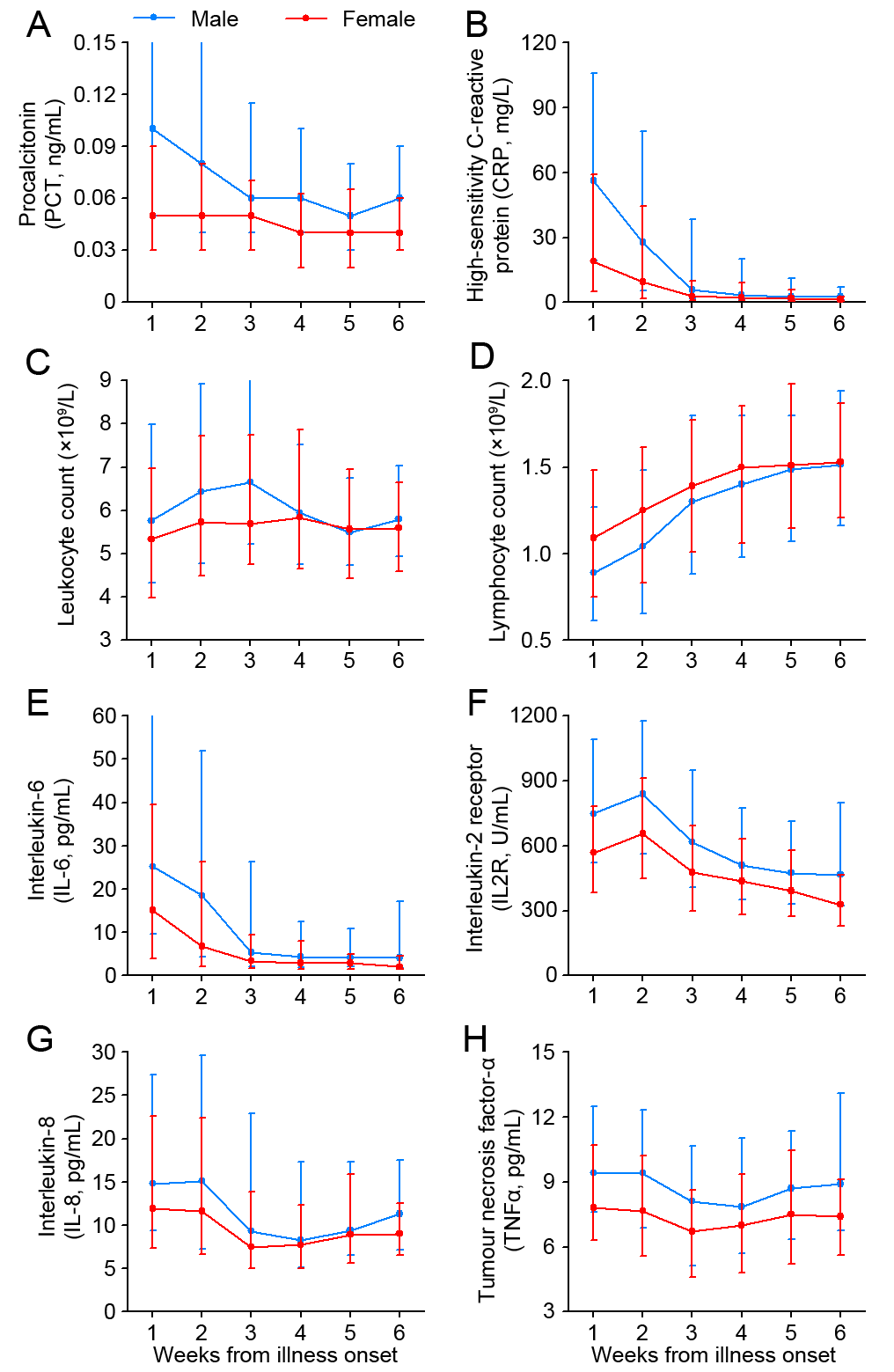


Figure shows temporal changes in the median and IQR of procalcitonin (A), high sensitivity C-reactive protein (B), leukocyte count (C), lymphocyte count (D), interleukin-6 (E), interleukin-2R (F), interleukin-8 (G) and tumor necrosis factor-α (H). The differences between males and females were significant for all timepoints shown, except for week 4–6 after illness onset for leukocyte count and interleukin-8, and week 5–6 for lymphocyte count.

**Supplement figure 3. Performance of SOFAmax in predicting the mortality of coronavirus disease 2019 patients.**


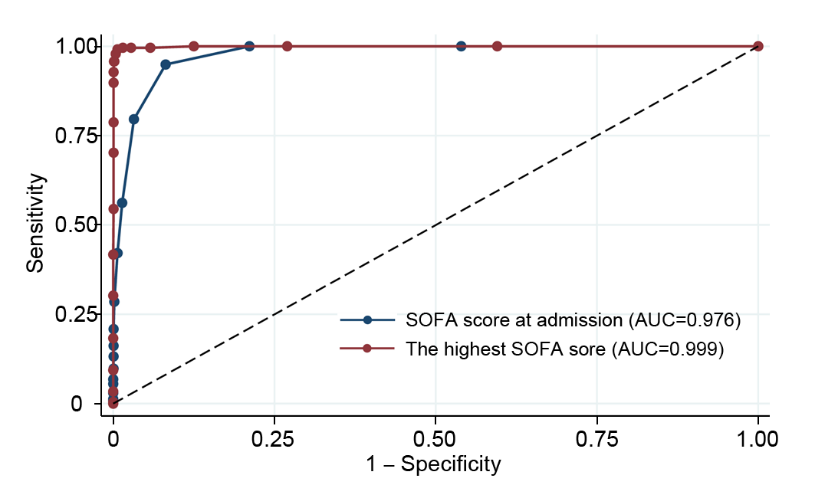


Receiver operating characteristic curve for the predicted value of SOFA score at admission and the SOFA max for the mortality of coronavirus disease 2019 patients in this study.

AUC=area under the curve. SOFA=Sequential Organ Failure Assessment. SOFAmax=the highest SOFA score during patients’ hospitalization.

**Supplement Figure 4: Different effects of age on developing a higher SOFAmax score for male and female patients.**


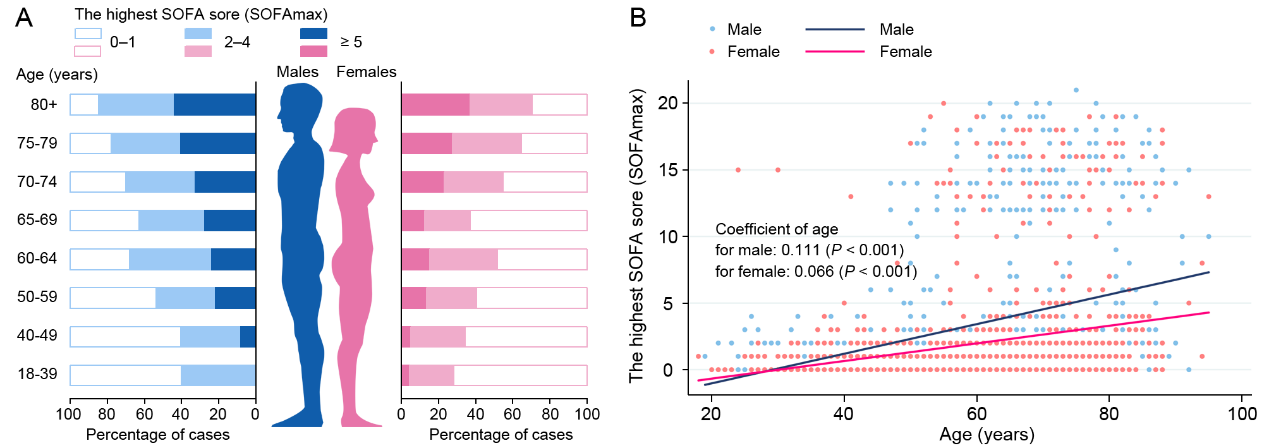


A. The proportions of male and female patients with SOFAmax =0–1, SOFAmax =2–4 and SOFAmax ≥5 in different age groups were shown.

B. The relationship between SOFAmax and age for male and female patients was analyzed using linear regression model containing age, sex, and the interaction term of age and sex. The interaction between age and sex was statistical significance (P =0.001), thus the different correlation coefficients and P values of age in male and female population were reported separately.

SOFAmax=maximum Sequential Organ Failure Assessment score during hospitalization.

**Supplement figure 5: Difference of inflammation biomarkers and cytokines between female and male patients with COVID-19.**


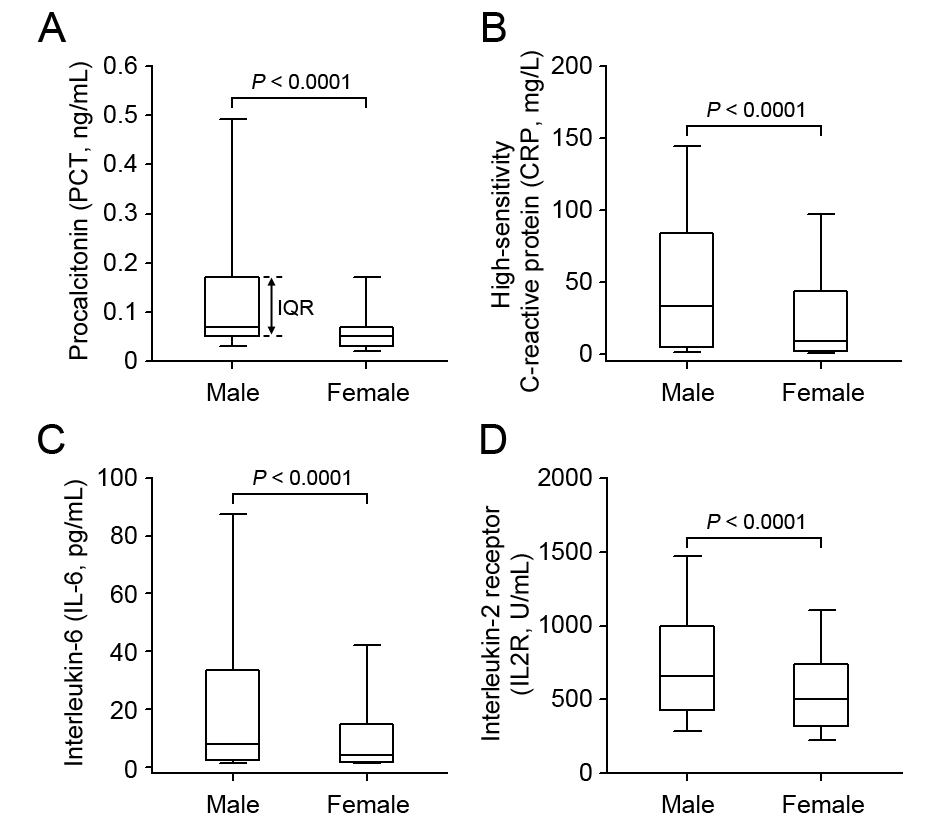


Figure show in the median and IQR of procalcitonin (A), high-sensitivity C-reactive protein (B), interleukin-6 (C), and interleukin-2 receptor (D). COVID-19=coronavirus disease 2019.

**Supplement Tables**

**Supplement table 1. Clinical characteristics of male and female patients at admission.**

|  | Total (n=2044) | Male (n=1000) | Female (n=1044) | *p value* |
| --- | --- | --- | --- | --- |
| Age, years | 62 (51–70) | 63 (50–70) | 62 (52–69) | *0.743* |
| ≥ 60y | 1177/2044 (57.58%) | 571/1000 (57.10%) | 606/1044 (58.05%) | *0.665* |
| Temperature, °C | 38.3 (37.5–39) | 38.5 (37.8–39) | 38 (37.1–38.8) | *<0.0001* |
| Respiratory rate, per min | 21 (20–24) | 21 (20–24) | 20 (20–23) | *0.008* |
| ≥24 breaths per min | 525/2039 (25.75%) | 279/997 (27.98%) | 246/1042 (23.61%) | *0.024* |
| Mean arterial pressure, mmHg | 96.67 (88.67–105.67) | 98 (90–106.67) | 95.33 (87.33–104.17) | *<0.0001* |
| <70 mmHg | 20/2041 (0.98%) | 8/998 (0.80%) | 12/1043 (1.15%) | *0.424* |
| SpO2, % | 95 (92–97) | 95 (91–97) | 96 (93–97) | *<0.0001* |
| ≤93% | 562/2038 (27.58%) | 325/997 (32.60%) | 237/1041 (22.77%) | *<0.0001* |
| SOFA score |  |  |  |  |
| 0-1 | 1427/2044 (69.81%) | 623/1000 (62.30%) | 804/1044 (77.01%) | *<0.0001* |
| 2-4 | 372/2044 (18.20%) | 216/1000 (21.60%) | 156/1044 (14.94%) |  |
| ≥5 | 245/2044 (11.99%) | 161/1000 (16.20%) | 84/1044 (8.05%) |  |
| Disease severity status |  |  |  |  |
| Moderate | 1087/2044 (53.18%) | 475/1000 (47.50%) | 612/1044 (58.62%) | *<0.0001* |
| Severe | 689/2044 (33.71%) | 349/1000 (34.90%) | 340/1044 (32.57%) |  |
| Critical | 268/2044 (13.11%) | 176/1000 (17.60%) | 92/1044 (8.81%) |  |
| **Onset Symptoms** | | | | |
| Fever | 1644/2041 (80.55%) | 853/998 (85.47%) | 791/1043 (75.84%) | *<0.0001* |
| Cough | 1478/2041 (72.86%) | 732/998 (73.35%) | 755/1043 (72.39%) | *0.626* |
| Sputum | 805/2041 (39.44%) | 390/998 (39.08%) | 415/1043 (39.79%) | *0.743* |
| Dyspnea | 870/2041 (42.63%) | 480/998 (48.10%) | 390/1043 (37.39%) | *<0.0001* |
| Fatigue | 736/2041 (36.06%) | 352/998 (35.27%) | 384/1043 (36.82%) | *0.467* |
| Diarrhea | 488/2041 (23.91%) | 223/998 (22.34%) | 265/1043 (25.41%) | *0.105* |
| Myalgia | 406/2041 (19.89%) | 195/998 (19.54%) | 211/1043 (20.23%) | *0.696* |
| Vomiting | 93/2041 (4.56%) | 40/998 (4.00%) | 53/1043 (5.08%) | *0.245* |
| Disorder in consciousness | 63/2041 (3.08%) | 40/998 (4.00%) | 23/1043 (2.20%) | *0.019* |
| **Comorbidities** |  |  |  |  |
| Patients with comorbidities, n | 1175/2039 (57.63%) | 592/996 (59.44%) | 583/1043 (55.90%) | *0.106* |
| Hypertension | 810/2039 (39.73%) | 403/996 (40.46%) | 407/1043 (39.02%) | *0.507* |
| Diabetes | 341/2039 (16.72%) | 190/996 (19.08%) | 151/1043 (14.48%) | *0.005* |
| Coronary heart disease | 199/2039 (9.76%) | 104/996 (10.44%) | 95/1043 (9.11%) | *0.340* |
| Chronic liver disease | 78/2039 (3.82%) | 47/996 (4.71%) | 31/1043 (2.97%) | *0.041* |
| Carcinoma | 63/2039 (3.09%) | 33/996 (3.31%) | 30/1043 (2.88%) | *0.569* |
| Chronic kidney disease | 32/2039 (1.57%) | 18/996 (1.81%) | 14/1043 (1.34%) | *0.398* |
| Chronic obstructive pulmonary disease | 18/2039 (0.88%) | 13/996 (1.31%) | 5/1043 (0.48%) | *0.046* |
| **Laboratory findings** | | | | |
| White blood cell count, ×10^9^ per L | 5.69 (4.41–7.38) | 5.87 (4.51–7.72) | 5.51 (4.345–7.125) | *0.001* |
| >10 | 192/2042 (9.40%) | 113/998 (11.32%) | 79/1044 (7.57%) | *0.004* |
| Lymphocyte count, ×10^9^ per L | 1.12 (0.77–1.56) | 1.03 (0.68–1.46) | 1.25 (0.85–1.64) | *<0.0001* |
| <0.8 | 555/1041 (27.19%) | 327/997 (32.80%) | 228/1044 (21.84%) | *<0.0001* |
| Hemoglobin, g/L | 126 (116–137) | 135 (125–144) | 120 (111–128) | *<0.0001* |
| Anemia | 392/2041 (19.21%) | 169/997 (16.95%) | 223/1044 (21.36%) | *0.011* |
| Platelet count, ×10^9^ per L | 222 (167–291.5) | 207 (156–277) | 232 (178–303) | *<0.0001* |
| <100 | 96/2036 (4.72%) | 58/993 (5.84%) | 38/1043 (3.64%) | *0.019* |
| Alanine aminotransferase, U/L | 22 (14.5–37) | 29 (19–46) | 18 (12–28) | *<0.0001* |
| >40 | 446/2044 (21.82%) | 307/1000 (30.70%) | 139/1044 (13.31%) | *<0.0001* |
| Aspartate aminotransferase, U/L | 25 (19–38) | 30 (21–44) | 22 (17–32) | *<0.0001* |
| >40 | 430/2036 (21.12%) | 286/995 (28.74%) | 144/1041 (13.83%) | *<0.0001* |
| Albumin, g/L | 35.5 (32–39.4) | 34.6 (31.2–38.4) | 36.4 (33–39.9) | *<0.0001* |
| ≤30 | 292/2041 (14.31%) | 172/998 (17.23%) | 120/1043 (11.51%) | *<0.0001* |
| Total bilirubin, μmol/L | 8.7 (6.5–12.1) | 10 (7.5–13.7) | 7.8 (5.7–10.4) | *<0.0001* |
| ≥20 | 97/2041 (4.75%) | 68/998 (6.81%) | 29/1043 (2.78%) | *<0.0001* |
| Lactate dehydrogenase, U/L | 262 (203–351) | 283 (213–396) | 246 (195–318) | *<0.0001* |
| ≥245 | 1156/2038 (56.72%) | 634/995 (63.72%) | 522/1043 (50.05%) | *<0.0001* |
| Blood urea nitrogen, μmol/L | 4.4 (3.4–5.8) | 4.9 (3.8–6.6) | 4 (3.1–5.1) | *<0.0001* |
| ≥10 | 144/2044 (7.05%) | 97/1000 (9.70%) | 47/1044 (4.50%) | *0.017* |
| Creatinine, μmol/L | 68 (57–85) | 81 (70–94) | 58 (51.5–67) | *<0.0001* |
| ≥110 | 161/2044 (7.88%) | 119/1000 (11.90%) | 42/1044 (4.02%) | *<0.0001* |
| Uric acid, μmol/L | 255.25 (196.2–322) | 277 (215.9–347.55) | 234.75 (183–291) | *<0.0001* |
| Prothrombin time, s | 13.8 (13.3–14.5) | 14 (13.4–14.7) | 13.7(13.2–14.3) | *<0.0001* |
| ≥17 | 75/2007 (3.74%) | 50/982 (5.09%) | 25/1025 (2.44%) | *0.002* |
| Activated partial thromboplastin time, s | 38.8 (35.8–42.7) | 39.6 (36.5–43.9) | 38.2 (35.3–41.6) | *<0.0001* |
| ≥52 | 76/1839 (4.13%) | 45/892 (5.04%) | 31/947 (3.27%) | *0.056* |
| D-Dimer, μg/L | 0.73 (0.35–1.74) | 0.83 (0.385–1.975) | 0.64 (0.32–1.47) | *0.0001* |
| >1 | 794/1969 (40.33%) | 426/960 (44.38%) | 368/1009 (36.47%) | *0.050* |
| High-sensitivity cardiac troponin I, pg/mL | 5.9(2.2–19.6) | 7.35(2.6–24.3) | 4.5(1.9–13.7) | *0.001* |
| >34·2 for male, >15·6 for female | 244/1725 (14.14%) | 106/797 (13.30%) | 138/928 (14.87%) | *<0.0001* |
| NT-proBNP, pg/mL | 119 (44.5–353) | 119 (38–406) | 119 (51–322) | *0.032* |
| >450 | 330/1608 (20.52%) | 180/777 (23.17%) | 150/831 (18.05%) | *0.011* |
| C-reactive protein, mg/L | 17.15 (2.5–64.8) | 33.3 (4.8–83.2) | 8.8 (1.7–43.2) | *<0.0001* |
| >10 | 1110/1936 (57.33%) | 636/953 (66.74%) | 474/983 (48.22%) | *<0.0001* |
| Erythrocyte sedimentation rate, mm/h | 32 (15–58.5) | 31 (14–58) | 32 (16–60) | *0.149* |
| Ferritin, ug/L | 554.7 (313.8–1067.3) | 808 (488.9–1436.8) | 387.45 (207.4–736) | *<0.0001* |
| >600 | 535/1159 (46.16%) | 380/589 (64.52%) | 155/570 (27.19%) | *<0.0001* |
| Procalcitonin, ng/mL | 0.06 (0.04–0.11) | 0.07 (0.05–0.17) | 0.05 (0.03–0.07) | *<0.0001* |
| ≥0.25 | 219/1749 (12.52%) | 149/868 (17.17%) | 70/881 (7.95%) | *<0.0001* |
| IL-2R, U/mL | 570 (363–871) | 657 (426–993) | 500 (319.5–736) | *<0.0001* |
| >710 | 589/1654 (35.61%) | 365/810 (45.06%) | 224/844 (26.54%) | *<0.0001* |
| IL-6, pg/mL | 5.37 (2.02–22.45) | 8.11 (2.63–33.59) | 4.035 (1.65–14.87) | *<0.0001* |
| ≥14 | 547/1663 (32.89%) | 327/813 (40.22%) | 220/850 (25.88%) | *<0.0001* |
| IL-8, pg/mL | 10.7 (6.1–20.9) | 11.85 (6.5–24.3) | 10 (5.7–18) | *<0.0001* |
| ≥62 | 110/1653 (6.65%) | 63/810 (7,78%) | 47/843 (5.58%) | *0.072* |
| TNFα, pg/mL | 7.7 (5.8–10.3) | 8.5 (6.5–11.2) | 7.1 (5.3–9.4) | *<0.0001* |
| ≥8.1 | 762/1651 (46.15%) | 454/809 (56.12%) | 308/842 (36.58%) | *<0.0001* |
| **Imaging features** | | | | |
| Consolidation | 254/1981 (13.36%) | 132/961 (14.52%) | 122/1020 (12.30%) | *0.155* |
| Ground-glass opacity | 1030/1903 (54.13%) | 488/910 (53.63%) | 542/993 (54.58%) | *0.676* |
| Bilateral pulmonary infiltration | 1833/1901 (92.53%) | 902/909 (93.86%) | 931/992 (91.27%) | *0.029* |

Data are median (IQR), n (%) or n/N (%). SOFA=Sequential Organ Failure Assessment. NT-proBNP=N-terminal pro B-type natriuretic peptide. IL2R=interleukin-2 receptor. IL-6=interleukin-6. IL-8=interleukin-8. IL-10= interleukin-10. TNF-α=tumor necrosis factor-α. p values were calculated by χ² test or Fisher’s exact test, as appropriate.

**Supplement table 2. Treatments and outcomes of female and male patients.**

|  | Total (n=2044) | Male (n=1000) | Female (n=1044) | *p value* |
| --- | --- | --- | --- | --- |
| **Treatment** |  |  |  |  |
| Antiviral treatments |  |  |  |  |
| Arbidol hydrochloride, n | 1653/2044 (80.87%) | 819/1000 (81.90%) | 834/1044 (79.89%) | *0.247* |
| Duration of use, days | 9 (6–12) | 9 (6–12) | 9 (6–12) | *0. 916* |
| Oseltamivir, n | 605/2044 (29.60%) | 304/1000 (30.40%) | 301/1044 (28.83%) | *0.437* |
| Duration of use, days | 6 (3–10) | 6 (3–9) | 6 (3–10) | *0. 402* |
| Ribavirin, n | 191/2044 (9.34%) | 99/1000 (9.90%) | 92/1044 (8.81%) | *0.398* |
| Duration of use, days | 5 (5–8) | 5 (1–8) | 5 (0–7.5) | *0. 973* |
| Aluvia, n | 422/2038 (20.71%) | 202/996 (20.28%) | 220/1042 (21.11%) | *0.643* |
| Duration of use, days | 8 (4–11) | 8 (4–11) | 8 (4–11) | *0.898* |
| Interferon, n | 215/2042 (10.53%) | 107/999 (10.71%) | 108/1043 (10.35%) | *0.793* |
| Duration of use, days | 3 (1–7) | 3 (1–7) | 3 (1–7) | *0.985* |
| Corticosteroids, n | 667/2044 (32.63%) | 371/1000 (37.10%) | 296/1044 (28.35%) | *<0.0001* |
| Duration of use, days | 7 (4–12) | 7 (4–12) | 7 (4–12) | *0.293* |
| Intravenous immunoglobin, n | 468/2044 (22.90%) | 256/1000 (25.60%) | 212/1044 (20.31%) | *0.004* |
| High-flow nasal cannula oxygen  Therapy, n | 75/1705 (4.40%) | 33/826 (4.00%) | 42/879 (4.78%) | *0.431* |
| Non-invasive mechanical  Ventilation, n | 117/1705 (6.86%) | 79/826 (9.56%) | 38/879 (4.32%) | *<0.0001* |
| Invasive mechanical  ventilation/ECMO, n | 129/1705 (7.57%) | 86/826 (10.41%) | 43/879 (4.89%) | *<0.0001* |
| Antibiotics, n | 1618/2043 (79.20%) | 810/999 (81.08%) | 808/1044 (77.39%) | *0.040* |
| Carbostyril | 1469/2043 (71.90%) | 745/999 (74.57%) | 724/1044 (69.35%) | *0.009* |
| Cephalosporin | 463/2043 (22.66%) | 226/999 (22.62%) | 237/1044 (22.70%) | *0.966* |
| Broad-spectrum antibiotics | 233/2043 (11.40%) | 139/999 (13.91%) | 94/1044 (9.00%) | *<0.0001* |
| **Outcomes** |  |  |  |  |
| In-hospital Death | 235/2044 (11.50%) | 156/1000 (15.60%) | 79/1044 (7.57%) | *<0.0001* |
| SOFAmax |  |  |  |  |
| 0-1 | 1321/2044 (64.63%) | 557/1000 (55.70%) | 764/1044 (73.18%) | *<0.0001* |
| 2-4 | 385/2044 (18.84%) | 227/1000 (22.70%) | 158/1044 (15.13%) |  |
| ≥5 | 338/2044 (16.54%) | 216/1000 (21.60%) | 122/1044 (11.69%) |  |
| ICU admission | 163/2044 (7.97%) | 107/1000 (10.70%) | 56/1044 (5.36%) | *<0.0001* |
| **Complication** |  |  |  |  |
| Sepsis | 711/2044 (34.78%) | 437/1000 (43.70%) | 274/1044 (26.25%) | *<0.0001* |
| Septic shock | 242/2044 (11.84%) | 159/1000 (15.90%) | 83/1044 (7.95%) | *<0.0001* |
| Respiratory failure | 273/2044 (13.36%) | 175/1000 (17.50%) | 98/1044 (9.39%) | *<0.0001* |
| ARDS | 446/2044 (21.82%) | 257/1000 (25.70%) | 189/1044 (18.10%) | *<0.0001* |
| Heart failure | 134/2041 (6.57%) | 87/999 (8.71%) | 47/1042 (4.51%) | *<0.0001* |
| Coagulopathy | 243/2044 (11.89%) | 157/1000 (15.70%) | 86/1044 (8.24%) | *<0.0001* |
| Acute cardiac injury | 309/1831 (16.88%) | 144/896 (16.07%) | 165/935 (17.65%) | *0.368* |
| Acute kidney injury | 250/2044 (12.23%) | 187/1000 (18.70%) | 63/1044 (6.03%) | *<0.0001* |
| Acute liver injury | 773/2044 (37.82%) | 505/1000 (50.50%) | 268/1044 (25.67%) | *<0.0001* |
| Hypoproteinemia | 474/2041 (23.22%) | 287/998 (28.76%) | 187/1043 (17.93%) | *<0.0001* |
| Secondary infection | 27/2044 (1.32%) | 12/1000 (1.20%) | 15/1044 (1.44%) | *0.639* |

Data are median (IQR), n (%) or n/N (%). ECMO=extracorporeal membrane oxygenation. SOFAmax=maximum Sequential Organ Failure Assessment score during hospitalization. ARDS=acute respiratory distress syndrome. ICU=intensive care unit. COVID-19=coronavirus disease 2019. p values were calculated by χ² test or Fisher’s exact test, as appropriate.

**Supplement table 3. prognosis analysis for female and male patients with COVID-19.**

|  | Univariable Analysis | | Multivariable Analysis* | |
| --- | --- | --- | --- | --- |
| Outcomes | OR for female (95%CI)  (males as reference 1) | *p value* | OR for female (95%CI)  (males as reference 1) | *p value* |
| In-hospital death | 0.443 (0.333, 0.590) | *<0.001* | 0.345 (0.218, 0.546) | *<0.001* |
| ICU admission | 0.473 (0.338, 0.662) | *<0.001* | 0.513 (0.328, 0.803) | *<0.001* |
| Sepsis | 0.458 (0.381, 0.552) | *<0.001* | 0.555 (0.429, 0.717) | *<0.001* |
| Sepsis shock | 0.457 (0.345, 0.605) | *<0.001* | 0.385 (0.249, 0.595) | *<0.001* |
| Respiratory failure | 0.488 (0.375, 0.636) | *<0.001* | 0.521 (0.354, 0.765) | *0.001* |
| ARDS | 0.639 (0.517, 0.790) | *<0.001* | 0.847 (0.630, 1.138) | *0.270* |
| Heart failure | 0.495 (0.343, 0.714) | *<0.001* | 0.712 (0.464, 10.92) | *0.119* |
| Coagulopathy | 0.482 (0.365, 0.637) | *<0.001* | 0.475 (0.333, 0.677) | *<0.001* |
| Acute cardiac injury | 1.119 (0.876, 1.430) | *0.368* | 2.049 (1.499, 2.800) | *<0.001* |
| Acute kidney injury | 0.279 (0.207, 0.377) | *<0.001* | 0.279 (0.192, 0.405) | *<0.001* |
| Acute liver injury | 0.339 (0.281, 0.408) | *<0.001* | 0.352 (0.285, 0.434) | *<0.001* |
| Hypoproteinemia | 0.541 (0.439, 0.667) | *<0.001* | 0.577 (0.438, 0.760) | *<0.001* |
| Secondary infection | 1.200 (0.559, 2.578) | *0.640* | .. | *..* |

OR=odds ratio. CI=confidence interval. ARDS=acute respiratory distress syndrome. ICU=intensive care unit. COVID-19=coronavirus disease 2019.

‥: Not applicable.

*: Age, number of comorbidities, white blood cell count, lymphocyte count, platelet count, creatinine, cTnI1, prothrombin time, alanine aminotransferase and female were chosen as the categorical variables for our multivariable logistic regression model. When the multivariable analysis was applied on coagulopathy, prothrombin time were no longer considered as variables in the logistic regression model, since the prothrombin time had collinearity with the occurrence of coagulopathy. For the same reason, alanine aminotransferase and Creatinine were not included in the multivariable logistic regression model for kidney and liver injuries, respectively.

**Supplement table 4. Risk factors associated with SOFAmax ≥5 in male and female patients revealed via univariable analysis.**

| **SOFAmax ≥5** | Univariable Analysis | | | |
| --- | --- | --- | --- | --- |
|  | Male | | Female | |
|  | OR (95% CI) | *p value* | OR (95% CI) | *p value* |
| **Characteristics** |  |  |  |  |
| Age, years | 1.065 (1.050, 1.081) | *<0.001* | 1.068 (1.048, 1.089) | *<0.001* |
| Presence of any comorbidities | 4.680 (3.057, 7.164) | *<0.001* | 2.934 (1.811, 4.754) | *<0.001* |
| CKD | 2.940 (1.124, 7.693) | *0.028* | 13.419 (4.558, 39.505) | *<0.001* |
| CLD | 1.404 (0.700, 2.814) | *0.339* | 1.835 (0.689, 4.889) | *0.225* |
| Carcinoma | 2.685 (1.296, 5.562) | *0.008* | 3.597 (1.556, 8.306) | *0.003* |
| Diabetes | 1.588 (1.086, 2.322) | *0.017* | 1.640 (0.980, 2.744) | *0.060* |
| SOFA score  ≥5 at admission | 66.398 (34.944, 126.166) | *<0.001* | 30.264 (16.429, 55.751) | *<0.001* |
| White blood cell count,  >10× 10^9^ per L | 10.220 (6.682, 15.631) | *<0.001* | 17.431 (10.421, 29.157) | *<0.001* |
| Platelet count,  <100× 10^9^ per L | 13.695 (7.567, 24.786) | *<0.001* | 16.341 (8.244, 32.391) | *<0.001* |
| Lymphocyte count,  <0.8× 10^9^ per L | 7.843 (5.475, 11.234) | *<0.001* | 8.216 (5.300, 12.737) | *<0.001* |
| High-sensitivity cardiac troponin I,  Male>34·2 pg/mL  Female>15·6 pg/mL | 32.297 (19.242, 54.208) | *<0.001* | 18.678 (11.559, 30.180) | *<0.001* |
| Prothrombin time,  ≥17s | 22.629 (11.060, 46.297) | *<0.001* | 45.383 (16.596, 124.101) | *<0.001* |
| Activated partial thromboplastin  time,  ≥52s | 5.766 (3.122, 10.651) | *<0.001* | 3.401 (1.476, 7.837) | *0.004* |
| D-Dimer,  >1 μg/L | 7.122 (4.764, 10.648) | *<0.001* | 13.246 (7.395, 23.727) | *<0.001* |
| IL-8,  Per 1 pg/ml increase | 1.008 (1.005, 1.011) | *<0.001* | 1.012 (1.008, 1.017) | *<0.001* |

.. ..: Not significant or not applicable. OR = Odds ratio. CI = Confidence interval. CHD = Coronary heart disease. COPD = Chronic obstructive pulmonary disease. IL2R = Interleukin-2 receptor. IL-6 = Interleukin-6. SOFAmax = Maximum Sequential Organ Failure Assessment score during hospitalization.

**Supplement table 5. Risk factors associated with SOFAmax ≥5 in male and female patients revealed via multivariable analysis.**

| **SOFAmax ≥5** | Multivariable Analysis | | | |
| --- | --- | --- | --- | --- |
|  | Male | | Female | |
|  | OR (95% CI) | *p value* | OR (95% CI) | *p value* |
| **Characteristics** |  |  |  |  |
| Hypertension | 2.576 (1.330, 4.987) | *0.005* | .. .. | *.. ..* |
| Procalcitonin,  Per 1 ng/mL increase | 1.754 (1.059, 2.903) | *0.029* | 3.533 (1.343, 9.292) | *0.011* |
| IL-6,  Per 1 pg/mL increase | 1.012 (1.006, 1.018) | *<0.001* | 1.015 (1.004, 1.025) | *0.006* |
| IL-2R  >710 U/mL | .. .. | *.. ..* | 3.361 (1.536, 7.358) | *0.002* |
| Age, years | 1.033 (1.006, 1.061) | *0.018* | 1.050 (1.017, 1.085) | *0.003* |
| Presence of any comorbidities | ‥ .. | *‥ ..* | ‥ .. | *‥ ..* |
| CKD | 6.309 (0.930, 42.795) | *0.059* | 5.359 (1.008, 28.490) | *0.003* |
| White blood cell count,  >10× 10^9^ per L | 4.540 (1.664, 12.383) | *0.003* | 4.117 (1.582, 10.715) | *0.004* |
| Platelet count,  <100× 10^9^ per L | 11.458 (3.982, 32.974) | *<0.001* | 6.298 (1.823, 21.763) | *0.004* |
| Lymphocyte count,  <0.8× 10^9^ per L | 4.427 (2.302, 8.512) | *<0.001* | .. .. | *.. ..* |
| High-sensitivity cardiac troponin I,  Male>34·2 pg/mL  Female>15·6 pg/mL | 6.195 (2.925, 13.120) | *<0.001* | 4.421 (2.070, 9.441) | *<0.001* |
| D-Dimer,  >1 μg/L | 2.167 (1.094, 4.294) | *0.027* | .. .. | *.. ..* |
| IL-8,  Per 1 pg/ml increase | .. .. | *.. ..* | 1.009 (1.002, 1.016) | *0.014* |

.. ..: Not significant or not applicable. OR = Odds ratio. CI = Confidence interval. CHD = Coronary heart disease. COPD = Chronic obstructive pulmonary disease. IL2R = Interleukin-2 receptor. IL-6 = Interleukin-6. SOFAmax = Maximum Sequential Organ Failure Assessment score during hospitalization.

**Reference**

1. Chen T, Wu D, Chen H, et al. Clinical characteristics of 113 deceased patients with coronavirus disease 2019: retrospective study. Bmj. 2020:m1091.

2. Pan A, Liu L, Wang C, et al. Association of Public Health Interventions With the Epidemiology of the COVID-19 Outbreak in Wuhan, China. JAMA. 2020.

3. National Health Commission of the People’s Republic of China. Interim diagnosis and treatment of 2019 novel coronavirus pneumonia. 7th ed. March 3, 2020. Accessed April 14, 2020. <http://www.nhc.gov.cn/yzygj/s7653p/202003/46c9294a7dfe4cef80dc7f5912eb1989.shtml>.

4. Zhou F, Yu T, Du R, et al. Clinical course and risk factors for mortality of adult inpatients with COVID-19 in Wuhan, China: a retrospective cohort study. The Lancet. 2020;395(10229):1054-1062.

5. Kdigo A. KDIGO clinical practice guideline for acute kidney injury. Kidney Int Suppl. 2012;2:1-138.

6. Antonelli M. Acute respiratory distress syndrome: the Berlin Definition. 2012.

7. Ponikowski P, Voors AA, Anker SD, et al. 2016 ESC Guidelines for the diagnosis and treatment of acute and chronic heart failure: The Task Force for the diagnosis and treatment of acute and chronic heart failure of the European Society of Cardiology (ESC)Developed with the special contribution of the Heart Failure Association (HFA) of the ESC. Eur Heart J. 2016;37(27):2129-2200.
